# Routine CSF Analysis in Coccidioidomycosis Is Not Required

**DOI:** 10.1371/journal.pone.0064249

**Published:** 2013-05-22

**Authors:** George Thompson, Sharon Wang, Robert Bercovitch, Michael Bolaris, Dane Van Den Akker, Sandra Taylor, Rodrigo Lopez, Antonio Catanzaro, Jose Cadena, Peter Chin-Hong, Brad Spellberg

**Affiliations:** 1 Coccidioidomycosis Serology Laboratory, Department of Medical Microbiology and Immunology, University of California Davis, Davis, California, United States of America; 2 Division of Infectious Diseases, Department of Internal Medicine, University of California Davis Medical Center, Sacramento, California, United States of America; 3 Division of Pulmonary and Critical Care Medicine, Department of Internal Medicine, University of California San Diego, San Diego, California, United States of America; 4 Division of Pediatric Infectious Diseases, Los Angeles Biomedical Research Institute at Harbor-University of California Los Angeles (UCLA) Medical Center, Torrance, California, United States of America; 5 Division of General Internal Medicine, Los Angeles Biomedical Research Institute at Harbor- University of California Los Angeles (UCLA) Medical Center, Torrance, California, United States of America; 6 Division of Biostatistics, Department of Public Health Sciences, University of California Davis, Davis, California, United States of America; 7 Division of Infectious Diseases, Department of Internal Medicine, University of Texas Health Sciences Center at San Antonio, San Antonio, Texas, United States of America; 8 Department of Internal Medicine, South Texas Veterans Health Care System, San Antonio, Texas, United States of America; 9 Division of Infectious Diseases, Department of Interal Medicine, University of California San Francisco, San Francisco, California, United States of America; 10 David Geffen School of Medicine at UCLA, Los Angeles, California, United States of America; University of Medicine & Dentistry of New Jersey - New Jersey Medical School, United States of America

## Abstract

Although routinely done, there has been no evaluation of the utility of performing routine cerebrospinal fluid (CSF) examination in patients with active coccidioidomycosis and high complement fixation (IgG) antibody titers or other risk factors for disseminated infection. In our review 100% of patients diagnosed with coccidioidal meningitis had at least one sign or symptom consistent with infection of the central nervous system, headache was present in 100% of those with meningitis, while no patients without signs/symptoms of CNS infection were found to have coccidioidal meningitis, irrespective of antibody titers or other risk factors. Thus routine lumbar puncture may be unnecessary for patients with coccidioidomycosis who lack suggestive clinical symptoms.

## Introduction

Coccidioidomycosis refers to the spectrum of disease caused by the dimorphic fungi *Coccidioides immitis* and *Coccidioides posadasii*. Over 150,000 patients are infected yearly and the incidence continues to increase [1], [2]. Pulmonary infection is the most common clinical manifestation, however the spectrum of disease ranges from asymptomatic exposure with subsequent immunity, to severe and life-threatening disseminated disease [1], [3].

Serum coccidioidal complement fixation (CF) titers greater than 1∶16 are predictive of an increased likelihood of disseminated infection [4], [5], and although skin and osteomyelitis/synovitis are the most common manifestations of dissemination, meningitis remains the most feared complication – occurring in nearly half of all patients with extrapulmonary coccidioidomycosis [6], [7]. Unfortunately, no data are available to determine the frequency of clinically silent central nervous system (CNS) involvement. Thus, lumbar puncture (LP) with cerebrospinal fluid (CSF) analysis is often performed on patients with high CF titers or other risk factors for meningitis even in the absence of CNS symptoms.

The rationale for this practice is based on the high morbidity observed in coccidioidal meningitis, the need for life-long antifungal therapy if coccidioidal meningitis is diagnosed, and the potential for earlier diagnosis to potentially avoid untoward outcomes [8]. However, it remains unclear how commonly patients with CNS coccidioidomycosis present without relevant symptoms, and anecdotally we have found routine LPs unhelpful in the absence of patient symptoms compatible with infection of the CNS. Since this practice subjects patients to potentially harmful and unneeded diagnostic testing, we sought to determine the utility of this approach in both patients with and without CNS symptoms in a multi-center, retrospective study.

## Materials and Methods

This study was approved by the Institutional Review Board of UC-Davis, UC-San Francisco, Harbor-UCLA, UC-San Diego, and UT-San Antonio. A waiver of consent was granted given the retrospective nature of the project. Cases were identified by searching hospital databases from 2005–2010 for *ICD9-CM* codes 114.0–114.9. Patient charts were manually reviewed and all patients with proven coccidioidomycosis who underwent CSF analysis were included for analysis. Patients were excluded if CSF analysis was not performed, or if the patient had a known diagnosis of CNS coccidioidal infection prior to presentation to one of the above mentioned institution(s), or charts were miscoded. Meningitis was defined as the presence of coccidioidal precipitin (IgM) antibody, or CF (IgG) antibody in the CSF in accordance with existing EORTC/MSG guidelines [9].

Clinical variables collected included demographic data, patient symptoms and exam findings, serum coccidioidal antibody titers, and CSF results. Neurologic abnormalities were defined as headache, photophobia, focal deficits, seizures, neurologic abnormalities on examination or abnormal CNS radiographic studies. The presence or absence of each of 21 signs and symptoms (headache, fever, nuchal rigidity, photophobia, motor focal deficit, sensory deficit, recent seizures, altered mental status, papilledema, abnormal CNS imaging, gaze palsy, abnormal visual fields, facial palsy, left/right arm palsy/drift, left/right leg palsy/drift, ataxia, aphasia, abnormal sensation, and dysarthria) were abstracted from patient charts (modified from [10]).

Mantel-Haenszel’s test was used to evaluate the association between the presence of at least one sign or symptom and CNS infection and Woolf’s test was applied to test for homogeneity of odds ratios across institutions. P values <0.05 were considered statistically significant.

## Results

Patients with coccidioidomycosis who did not undergo CSF analysis were excluded. A total of 353 charts were reviewed, 249 with coccidioidomycosis. Of the reviewed charts, 58 coccidioidomycosis cases who underwent CSF analysis were included in this study. A large number of patients were thus excluded due to a lack of lumbar puncture given no clinical concern for meningitis at time of physician evaluation. No statistically significant differences were found in demographic or host risk factors between patient groups with or without coccidioidal meningitis (p>0.05) (Table 1).

**Table 1:** Baseline characteristics of 58 patients with suspected coccidioidal meningitis who underwent CSF evaluation.

		Found to have coccidioidalmeningitis (n = 14)	Patients without coccidioidal meningitis (n = 44)
Age, median years (range)		49 (25–78)	41 (19–69)
Male sex		12 (86%)	37 (84%)
Ethnicity			
Ethnicity			
	Hispanic	10	17
	Caucasian	2	11
	Black	0	10
	Asian/Pacific Islander	1	5
	Unknown	1	1
Immunosuppressed			
	HIV	7	16
	CD4<200	6	14
	Transplant	0	1
	Immunosuppressive medications	0	2
	Diabetes Mellitus	5	7
Concurrent coccidioidal infection*			
	Primary coccidioidal pneumonia	2	7
	Chronic Pulmonary¥	8	26
	Joint/Bone	2	8
	Cutaneous	0	15
	Fungemia	1	2
	Peritoneal disease	0	2

* Some patients had more than one type of coccidioidal infection.

¥Defined by positive sputum cultures or cavity for >6 months.

Nineteen patients were from northern California (UC Davis and UCSF), 21 from southern California (UCSD and UCLA) and 18 from Texas (UT). All patients included had proven coccidioidomycosis in accordance with the Mycosis Study Group-European Organization for the Research and Treatment of Cancer (MSG/EORTC) criteria [9]. The patients were predominantly male (84%) and Hispanic (47%). Other risk factors for coccidioidomycosis included infection with the human immunodeficiency virus (HIV) (41%), diabetes mellitus (21%) and receipt of immunosuppressive medications (4%). Interestingly, 17/58 (29.3%) patients underwent CSF evaluation despite no signs or symptoms attributable to CNS infection. Fourteen (24%) of the patients were diagnosed with coccidioidal meningitis. All 14 patients with meningitis had at least one symptom or sign related to CNS disease (Table 2). Among the symptoms reported, headache was the most common, occurring in 14/14 (100%) of patients with meningitis and only 18/44 (41%) of those without meningitis (Table 2). The presence of a headache was significantly associated with meningitis (P<0.001). The odds ratio of meningitis for patients with headaches to those without did not differ significantly across the three study areas (P = 0.629) and was estimated as 14.53 [95% C.I. 2.47–85.44]. Thus, 0% of patients [95% C.I. = 0−24%] had CSF evidence of meningitis in the absence of signs or symptoms. However, the presence of at least one symptom or sign related to CNS disease had poor positive predictive value and was not significantly associated with the diagnosis of meningitis (P = 0.229). Odds ratios did not significantly differ across the three study areas (P = 0.794).

**Table 2:** Clinical variables associated with CNS coccidioidomycosis.

Symptom	Number with CNS infection (n = 14)	Number without CNS Infection (n = 44)
Headache	14	18
Fever	5	13
Nuchal rigidity	3	3
Photophobia	2	3
Focal motor deficit	1	0
Focal sensory deficit	0	0
Recent seizures	0	2
Altered mental status	4	8
Papilledema	0	0
Abnormal CNS imaging	7	5
Abnormal visual fields	0	1
Ataxia	2	0

Pooled estimates for the sensitivity, specificity, likelihood ratio positive and likelihood ratio negative of 1) at least one CNS disease sign or symptom and 2) headache alone as a predictors for meningitis are provided in Table 3. Sensitivities were very high, estimated as 1.00, indicating that all patients with meningitis exhibited some sign or symptom of CNS disease but the low specificities indicate that many patients without meningitis also present with these symptoms. Using the pooled estimates for sensitivity and specificity, we estimated the positive and negative predictive values at prevalences of 0.15 and 0.3, which bounds the range observed in this study for three study sites (Northern California: 0.18 [3/17]; Southern California: 0.29 [6/21]; Texas: 0.25 [5/20]). Both the presence of at least one CNS disease sign or symptom or headache alone, had high negative predictive values (Table 4). The high negative predictive values indicate that patients with meningitis rarely fail to exhibit symptoms.

**Table 3:** Pooled estimates (95% confidence limits) for sensitivity, specificity, likelihood ratio positive, likelihood ratio negative for 1) at least 1 CNS disease sign or symptom and 2) headache alone as predictors of meningitis.

	At least 1 sign/symptom	Headache alone
Sensitivity	1.00 [0.77, 1.00]	1.00 [0.77, 1.00]
Specificity	0.25 [0.13, 0.40]	0.59 [0.43, 0.74]
Likelihood Ratio Positive	1.25 [0.99, 1.59]	2.30 [1.55, 3.42]
Likelihood Ratio Negative	0.33 [0.07, 1.59]	0.15 [0.03, 0.68]

**Table 4:** Positive and negative predictive values (95% confidence limits) for 1) at least 1 CNS disease sign or symptom and 2) headache alone as predictors of meningitis for prevalences of 0.15 and 0.30.

	At least 1 sign/symptom		Headache only	
Prevalence	Positive Predictive Value	Negative Predictive Value	Positive Predictive Value	Negative Predictive Value
0.15	0.19 [0.09, 0.33]	1 [0.73, 100]]	0.30 [0.14, 0.50]	1 [0.88, 1]
0.30	0.36 [0.23, 0.52]	1 [0.69, 100]	0.51 [0.33, 0.69]	1 [0.85, 1]

A sub-group analysis of the 24 patients with HIV was conducted. In this group 8 patients (33%) were ultimately diagnosed with coccidioidal meningitis (6 with CD4<200 cells/µL). All patients with HIV and coccidioidal meningitis exhibited symptoms consistent with CNS infection (100% had headache) suggesting that even in immunocompromised patients most manifest symptoms consistent with CNS coccidioidomycosis.

**Figure 1:**
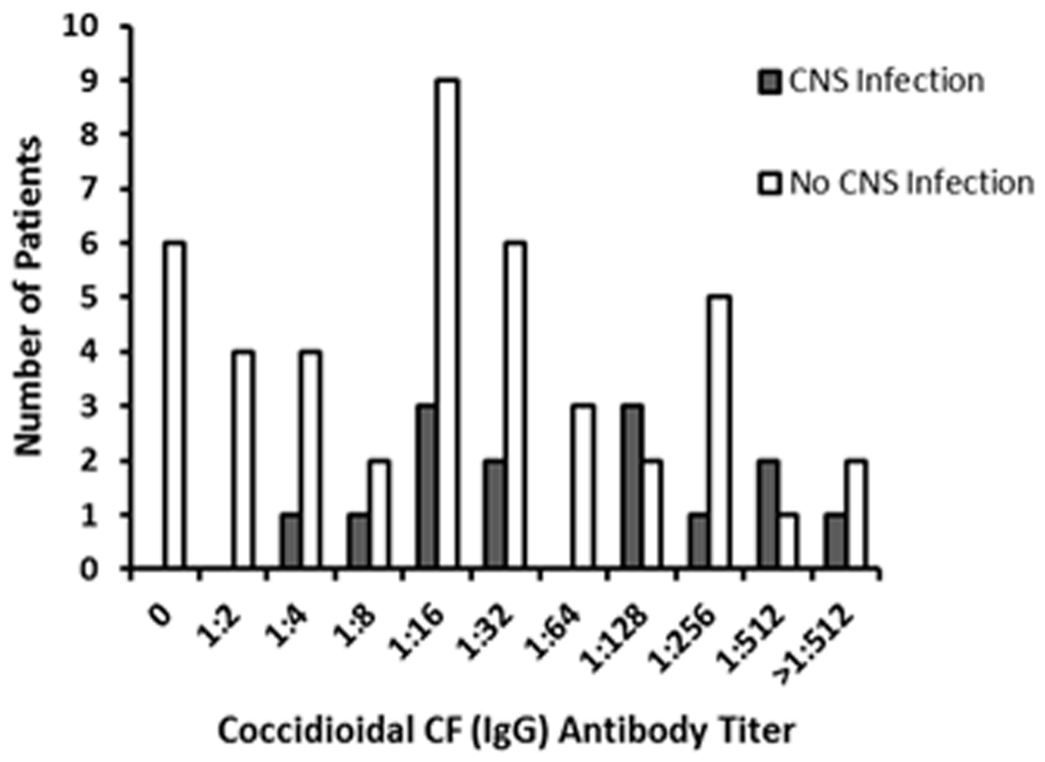
Coccidioidal CF (IgG) serum antibody titers and association with coccidioidal meningitis.

Coccidioidal complement fixation titers exhibited considerable variation between patients, and only 2 (3%) patients with coccidioidal meningitis exhibited antibody titers <1∶16. Patients without coccidioidal meningitis also exhibited a wide-range of CF antibody titers (range 0 - 1∶512) (Figure 1). No serum CF titer cut-off was useful at distinguishing patients with vs. without meningeal disease. Low titer coccidioidal CF antibody can be detected in the CSF of patients even in the absence of coccidioidal meningitis, however in our study coccidioidal CF antibody in the CSF was found only in those with coccidioidal meningitis (confirmation by concurrent changes in the CSF cell count, protein, and glucose parameters). No patient given a non-coccidioidal diagnosis had detectable coccidioidal antibody in their CSF.

## Discussion

Coccidioidomycosis is a common infection with a widely variable presentation. Sixty percent of patients exposed to *Coccidioides* spp. manifest no symptoms and are identified only after exhibiting skin test positivity [11]. The remaining 40% of patients exhibit disease ranging from subacute pulmonary symptoms with fever, cough, and night sweats to disseminated disease with meningitis. Due to concerns about missing occult meningitis in patients with high CF titers (e.g., >1∶16) or other predisposing conditions, LPs are often performed even in the absence of signs or symptoms referable to CNS infection. However, this practice exposes patients to the risks and costs of an invasive procedure with uncertain benefit.

Current IDSA coccidioidomycosis guidelines recommend CSF analysis in patients who develop progressively severe or persistent headaches, mental status changes or other meningeal signs [1] and are silent on CSF examination in the asymptomatic patient. In the authors’ experience, CSF examination is routinely performed on patients with elevated coccidioidal CF titers, those with underlying immunosuppression, or those with other risk factors favoring the development of disseminated disease (Filipino or of African descent, etc) [12]. However, we found no benefit in conducting LPs in patients with coccidioidomycosis who had no signs or symptoms referable to CNS infection, irrespective of CF titer, immune status, or ethnicity. Thus, our results suggest that routine LP may not be required in the absence of symptoms referable to CNS infection. Furthermore, because 2 of 14 patients with meningitis had serum CF titers <1∶16, it is important to rule out meningitis in patients with relevant signs or symptoms even if CF antibody titers are low.

The decision to perform a lumbar puncture has important therapeutic implications as unrecognized coccidioidal meningitis has been associated with unfavorable outcomes [13]. Although our results suggest an algorithmic approach to the care of patients with coccidioidomycosis is unlikely to be helpful - our results do suggest CSF analysis in the asymptomatic is often performed (29.3% of the patients in our study).

Prior series of coccidioidal meningitis have not specifically examined the frequency of symptoms attributable to CNS infection, although such literature is generally concordant with the current study. One prior study included patients with proven, probable, and possible coccidioidal meningitis and found headache in 77% of patients; no comment was made on the frequency of asymptomatic patients ultimately diagnosed with CNS infection [14]. Similar findings were reported by Mathisen et al among patients diagnosed with coccidioidal meningitis in both 1980 (headache in 79%, N = 31) and 2008 cohorts (77%, N = 30), however they likewise did not describe any asymptomatic patients with CNS infection [15]. In another cohort, all 13 patients with coccidioidal meningitis exhibited CNS symptoms consistent with infection of the CNS [13]. These prior reports suggest that coccidioidal meningitis in the absence of suggestive clinical signs or symptoms is rare, consistent with our results.

Strengths of our analysis included its multi-centered nature and availability of clinical data to confirm meningeal disease or not. This is the largest attempt to refine the approach to evaluation for coccidioidal meningitis. Limitations include the fact that the data were collected retrospectively and patients with recurrent disease were excluded. The number of HIV+ patients was small, and larger series are needed to validate our results in immunosuppressed populations. In addition, because we included referral centers, there may be a bias towards inclusion of patients with more severe disease. It is possible that patients with asymptomatic meningitis were excluded from inclusion due to a lack of CSF analysis, however we believe this event is unlikely as disease progression over time would have allowed for later diagnosis and it therefore seems unlikely meaningful numbers of patients with meningitis were excluded. Prospective evaluation of our findings would provide additional evidence for a symptom-based approach to management.

Additionally, our results examined patients only with a “new” diagnosis of coccidioidomycosis, and clinicians may encounter patients diagnosed with coccidioidomycosis at other facilities with little ancillary data available at the time of their evaluation. In this circumstance, some clinicians would opt to perform CSF examination prior to discontinuation of antifungal therapy. This circumstance was not specifically evaluated in our study and antifungal therapy may mask CNS symptoms with meningitis presenting only after discontinuation of antimicrobial agents.

In summary we advocate for a careful history and physical examination of patients with coccidioidomycosis rather than automatic evaluation of the cerebrospinal fluid. Until larger prospective databases are available to validate the current results, we support a thoughtful and individualized approach to evaluating the risk of dissemination and presenting symptomatology as a guide to management decisions.
